# Effects of Pristane Alone or Combined with Chloroquine on Macrophage Activation, Oxidative Stress, and Th1/Th2 Skewness

**DOI:** 10.1155/2014/613136

**Published:** 2014-07-06

**Authors:** Qiufang Ouyang, Ziyang Huang, Zhenhua Wang, Xiaoqing Chen, Jingqin Ni, Ling Lin

**Affiliations:** ^1^Cardiovascular Department, Second Affiliated Hospital and Second Clinical Medical College, Fujian Medical University, Zhongshan North Road 34, Quanzhou, Fujian 362000, China; ^2^Rheumatism Department, Second Affiliated Hospital and Second Clinical Medical College, Fujian Medical University, Zhongshan North Road 34, Quanzhou, Fujian 362000, China

## Abstract

We investigated the protective role of chloroquine against pristane-induced macrophage activation, oxidative stress, and Th1/Th2 skewness in C57BL/6J mice. Those mice were treated with pristane alone or combined with chloroquine. Hematological and biochemical parameters, macrophage phagocytic function, the oxidant/antioxidant index, cytokine for IFN-*γ*, TNF-*α*, IL-4, and IL-6, and the isotypes of IgG2a and IgG1 were determined. And the expression of T-bet/GATA-3 and IL-12/IL-10 mRNA in spleen were analyzed by real-time PCR. We found that pristane treatment for a period of 12 or 24 weeks triggered macrophage activation syndrome, characterized by hemophagocytosis in spleen and peripheral blood, enhanced lipid phagocytosis by peritoneal macrophages in vitro, erythropenia and leucopenia, increased anti-Smith, lactic dehydrogenase, triglyceride, and ferritin, as well as hypercytokinemia of IFN-*γ*, TNF-*α*, IL-4, and IL-6. In parallel, a significant increase in lipid peroxidation and a decrease in superoxide dismutase, glutathione, and catalase activity, as well as a skewed Th1/Th2 balance in spleen, were observed. However, chloroquine supplementation showed a remarkable amelioration of these abnormalities. Our data indicate that pristane administration induces macrophage activation, oxidative stress, and Th1/Th2 skewness, which can be attenuated by chloroquine.

## 1. Introduction

The hemophagocytic syndrome, also termed macrophage activation syndrome (MAS) in the context of autoimmune diseases, is a life-threatening disease of severe hyperinflammation. It is characterized by fever, splenomegaly, cytopenias affecting at least 2 of 3 lineages in the peripheral blood, hyperferritinemia, hypertriglyceridemia and/or hypofibrinogenemia, hemophagocytosis in the bone marrow, spleen, or lymph nodes, and so forth [1]. The pathogenesis of MAS is still inconclusive. It is reported that a skew toward T-helper (Th) 1 immune response has been observed in MAS patients [2]. The Th1 cells upon activation secrete proinflammatory cytokines: interleukin- (IL-) 12, interferon- (IFN-) *γ*, and tumor necrosis factor- (TNF-) *α*, which mainly activate macrophages to produce reactive oxygen species (ROS) and mediate innate immunity [3]. Convincing data suggested oxidative stress and the Th1 cytokine levels correlated with systemic lupus erythematosus (SLE) disease activity [4].

Pristane (2,6,10,14-tetramethylpentadecane, TMPD), an immune adjuvant, is widely adopted to establish SLE or rheumatoid arthritis model. Mounting evidence suggests that pristane can induce autoantibody production, arthritis, pulmonary capillaritis, and glomerulonephritis [5]. However, far too little attention has been paid to the effects of pristane on macrophage activation and its related oxidative stress and Th1/Th2 polarity.

The antimalarial drug chloroquine (CQ) is an acidotropic agent that passively diffuses into acidic organelles. It is well documented that CQ can inhibit autophagy as it raises the lysosomal pH, which leads to inhibition of both fusion of autophagosome with lysosome and lysosomal protein degradation [6]. Gong reported that chloroquine was effective in treating patients with infection-associated hemophagocytic syndrome [7]. However, the data on the effects of CQ on MAS in animal models is unavailable.

Therefore, this study was designed to investigate whether macrophage activation syndrome, oxidative stress, and Th1/Th2 imbalance could be triggered by pristane and to further explore the role of chloroquine in preventing pristane-induced abnormalities in those parameters associated with macrophage activation.

## 2. Materials and Methods

### 2.1. Experimental Animals and Design

All of the procedures and protocols were approved by the* Animal Care Committee of Fujian Medical University* and followed the guidelines of* Animal Management Rules of the Chinese Ministry of Health* (Document number 55, 2001). 48 female C57BL/6J mice aged 10 weeks were obtained from the Department of Laboratory Animal Science, Beijing University. All animals were kept under standardized lighting conditions (12 h light-dark cycle) and temperature (21 ± 1°C). Mineral water was administered ad libitum. They were divided into three main groups (*n* = 16) as follows: (1) control group: this group was intraperitoneally (i.p.) injected with 200 *μ*L saline, every 2 days for 12 or 24 weeks; (2) pristane-treated group: 12 h after a single i.p. injection of 0.5 mL pristane (Sigma-Aldrich, Louis, MO, USA), 200 *μ*L saline was i.p. administered, every 2 days for 12 or 24 weeks; (3) pristane+CQ treated group: this group received a single i.p. injection of 0.5 mL pristane, and then 30 mg/kg chloroquine (Sigma-Aldrich, Louis, MO, USA) was i.p. supplemented, every 2 days for a period of 12 weeks. At the end of the experimental period, animals were sacrificed and specimens including blood, spleen, liver, and bone marrow were collected.

### 2.2. Histology in Liver and Spleen

Hematoxylin and eosin (HE) staining was performed in liver and spleen paraffin sections to detect hemophagocytosis and inflammation. Meanwhile, immunohistochemistry (IHC) was performed to detect F4/80 (1 : 150, Abcam, Cambridge, UK) positive macrophages.

### 2.3. Blood and Bone Marrow Smear and Hematological Parameters of Erythrocytes

For morphological analysis, smears of peripheral blood and bone marrow were stained with Wright-Giemsa staining and examined with Olympus BX61 microscope. Twenty microliters of fresh blood was collected and applied to a fully automated hematological analyzer (Sysmex KX-21N, Japan) to determine the parameters including red blood cells (RBCs), hemoglobin (HGB), white blood cells (WBCs), platelet count, and reticulocyte count.

### 2.4. Detection of Macrophage Phagocytosis In Vitro with Oil Red O Staining

Peritoneal macrophages were obtained from mice treated with 12-week saline or pristane. The macrophages were cultured for 48 h and subsequently incubated with oxidized LDL (500 ng/mL) for 24 h. Cells in culture slides were fixed with 4% paraformaldehyde for 10 min followed by rinsing with PBS and then by 60% isopropanol. The cells were stained with 30 mg/mL oil red O (Sigma, St. Louis, MO, USA) in 60% isopropanol for 10 min and again washed with isopropanol and then with PBS. Finally, the cells were treated with Mayer's hematoxylin for 5 min to stain nuclei. Phagocytosis index (number of ingested foam cells per 100 macrophages) was determined.

### 2.5. Analysis of Oxidative Stress in Blood

The levels of reactive oxygen species (ROS) in blood smear were measured using the fluorescent probe dihydroethidium (DHE, Invitrogen, Carlsbad, CA, USA). Products of lipid peroxidation were estimated by measuring the concentration of malondialdehyde (MDA) expressed as thiobarbituric acid reactive substances (TBARS) as previously described [8]. Meanwhile, the antioxidant enzymes including superoxide dismutase (SOD), glutathione (GSH), and catalase (CAT) were analyzed. All these assay kits were from Nanjing Jiancheng Bioengineering.

### 2.6. Real-Time PCR for CD4^+^Th Cell Subsets in Spleen

Total RNA isolated from spleen tissues was treated with DNase I at 37°C for 30 min before reverse transcription was performed using a high capacity cDNA archive kit (TaKaRa, Japan). The PCR was performed with the MasterMix System (Roche, Basel, Switzerland). The sequences of primers for Th1-related genes (T-bet, IL-12) and Th2-related genes (GATA-3, IL-4) were detected on spleen tissues (Table 1). Relative quantification of target gene mRNA used the comparative ΔΔC_T_-method, normalized to an endogenous reference glyceraldehyde-3-phosphate dehydrogenase (GADPH). The expression of target genes was determined in triplicate from the standard curve.

### 2.7. ELISA for IFN-*γ*, TNF-*α*, IL-6, IL-4, IgG1, IgG2a, Ferritin, and Fibrinogen and Biochemical Detection for LDH and Triglyceride in Plasma

The cytokine concentration was determined using sandwich ELISA for interferon-*γ* (IFN-*γ*), tumor necrosis factor alpha (TNF-*α*), interleukin-4 (IL-4), and IL-6 in serum according to the instructions (all from eBioscience, San Diego, CA, USA), and so were IgG1 and IgG2a (all from Alpha Diagnostic). The levels of ferritin and fibrinogen (Assaypro, Missouri) were assayed by ELISA method. All the measurements were carried out in duplicate. The levels of lactate dehydrogenase (LDH) and triglyceride in plasma were assayed with an automatic biochemistry analyzer (Hitachi, Japan).

### 2.8. Western Blot Analysis for F4/80 Expression in Liver and Spleen

Western blot analysis was performed to determine the F4/80 (Abcam, diluted at 1 : 500) protein levels in the liver and spleen tissue. The intensity of the bands was quantified by densitometry. Blots are representative of at least three experiments showing the same results.

### 2.9. Statistical Analysis

All the values are expressed as the mean ± SE unless otherwise indicated. The group comparisons were performed with Student's *t*-test (2-sample test) or analysis of variance. The Mann-Whitney *U* test was used if the variance was not normally distributed. A *P* value of 0.05 was accepted as significant. The statistical analysis was performed using SPSS 17.0 software.

## 3. Results

### 3.1. Effects of Pristane Alone or Combined with Chloroquine on Hemophagocytosis, Extramedullary Hematopoiesis, and Macrophage Infiltration in Liver and Spleen

**Figure 1:**
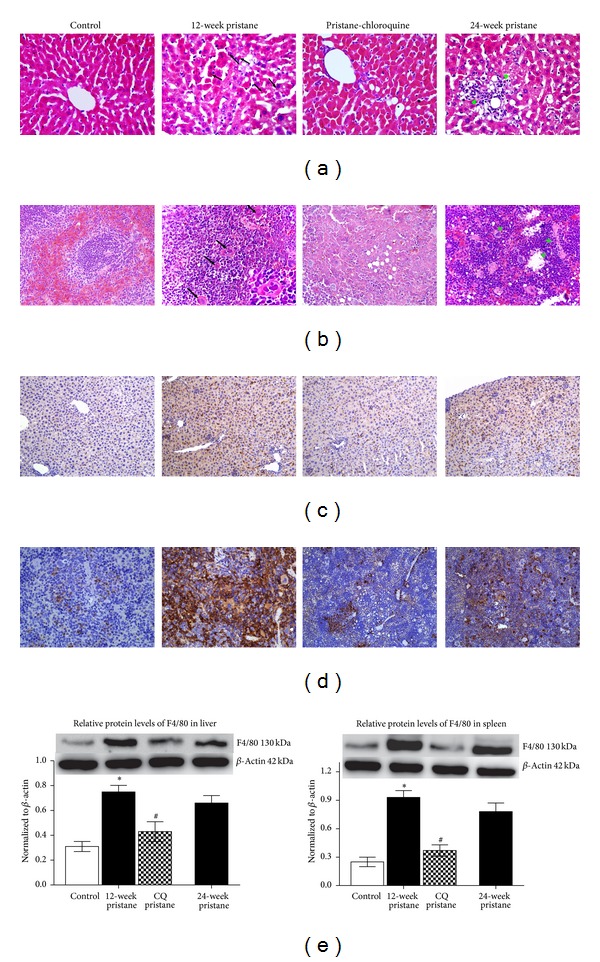
Hemophagocytosis, extramedullary hematopoiesis, and macrophage infiltration in liver and spleen. Hemophagocytosis (arrow), extramedullary hematopoiesis (arrow head) in liver and spleen were illustrated by HE staining ((a), (b)). The distribution and expression of F4/80 in liver and spleen were depicted with immunohistochemistry ((c), (d)) and western blotting (e), respectively. ×200; inset, ×400. CQ: chloroquine. Results were expressed as mean ± SE for eight animals. *P < 0.05 versus corresponding control mice, ^#^ *P* < 0.05 versus mice exposed to pristane for 12 weeks.

Control mice displayed normal structures of liver and spleen. And destroyed structure with lipid-vacuolated granulomas formation was observed after pristane injection. Splenomegaly (data not shown) and enhanced hemophagocytosis in liver and spleen were detected 12 weeks after pristane injection (Figure 1(b)), while, at 24 weeks after pristane injection, abundant extramedullary hematopoiesis including megakaryocytes, erythroid and granulocyte precursors was observed, with substantially increased megakaryocytes in spleen red pulp (Figure 1(d)). Alternatively, pristane induced an increased F4/80 protein expression in liver and spleen, as manifested by western blot analysis, which was further confirmed by IHC. Treatment with CQ for 12 weeks ameliorated hemophagocytosis and F4/80 positive macrophage infiltration.

### 3.2. Effects of Pristane Alone or Combined with Chloroquine on Cytological Characteristics in Blood and Bone Marrow

Considering that enhanced erythrophagocytosis and extramedullary hematopoiesis may be associated with anemia and bone marrow abnormity, cytology in blood and bone marrow was investigated. As compared to the control mice, the levels of RBCs, HGB, and WBCs dropped markedly at 12 weeks after pristane injection, which could be partially reversed by chloroquine treatment. At 24 weeks after pristane injection, those parameters were still lower than the age-matched controls (Table 2). Additionally, 29.6% mice displayed mild anemia and 18.5% moderate or severe anemia following exposure to pristane for 12 weeks. And the frequency of mild, moderate to severe anemia was 21.4% and 7.0%, respectively.

As compared with the age-matched control mice, the number of reticulocytes changed insignificantly at 12 weeks while it increased by 18.5% at 24 weeks after pristane administration. Meanwhile, significantly increased platelets were observed at both 12 and 24 weeks after pristane injection. Chloroquine treatment decreased platelets counts.

**Figure 2:**
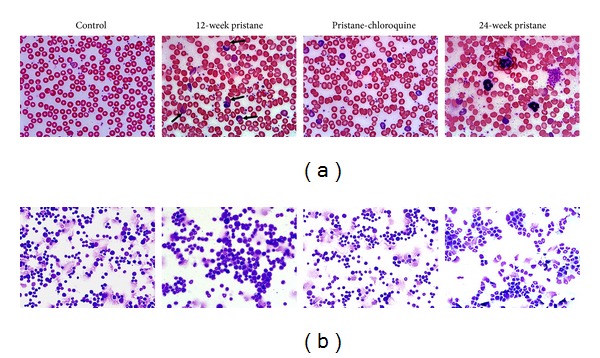
Effects of pristane alone or combined with chloroquine on cytological characteristics in blood and bone marrow. Wright-Giemsa staining was performed in peripheral blood (a) and bone marrow (b) smears. Hemophagocytosis (arrows), poikilocytosis, polychromasia, hypochromia, and anisocytosis of erythrocytes were observed in blood smear of pristane-treated mice. Bone marrow cytology depicted hypocellularity in erythroid progenitors, poikilocytosis, and nonerythroid lineage clonal expansion following treatment with pristane for 12 weeks or 24 weeks. And the severity of poikilocytosis was attenuated in the presence of chloroquine.

For morphological analysis, abnormal erythrocyte morphology such as stomatocytes, acanthocytes, schistocytes, and dacryocytes could be observed following pristane injection. And the severity of poikilocytosis was attenuated in the presence of chloroquine (Figure 2). Hemophagocytosis was also detected on peripheral blood smear. Concurrently, the bone marrow cells displayed clonal expansion in non-erythroid lineage progenitors and hypoplasia in erythroid progenitors. The myeloid/erythroid (M/E) ratio was significantly elevated following exposure to pristane for 12 weeks (4.53 ± 0.92 versus 2.12 ± 0.45) or 24 weeks (3.71 ± 0.78 versus 2.33 ± 0.51), as compared to their corresponding controls. The severity of poikilocytosis and the ratio of M/E were attenuated in the presence of chloroquine. Contrastingly, peripheral blood and bone marrow smears from control mice showed normal RBC morphology.

### 3.3. Chloroquine Inhibited Macrophage Phagocytic Function Induced by Pristane In Vitro

**Figure 3:**
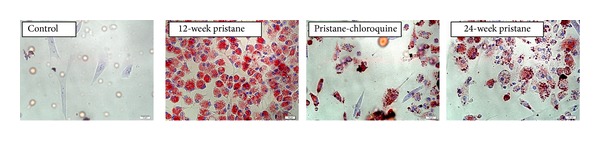
Macrophage phagocytic function revealed by oil red O staining. Peritoneal macrophages were exposed to 500 ng/mL ox-LDL for 24 h.

To further confirm the effects of pristane on the macrophage phagocytic function, peritoneal macrophages were collected to detect their lipid phagocytic activity (Figure 3). As compared to control mice, the phagocytic index was significantly higher after exposure to pristane for 12 weeks (95.2 ± 7.2% versus 35.4 ± 5.1%, *P* < 0.01) or for 24 weeks (78.6 ± 8.4% versus 31.7 ± 4.9%, *P* < 0.01). Phagocytosis was inhibited in peritoneal macrophages of chloroquine treated mice (65.9 ± 9.3%).

### 3.4. Enhanced Oxidative Stress Induced by Pristane Was Ameliorated by CQ Treatment

**Figure 4:**
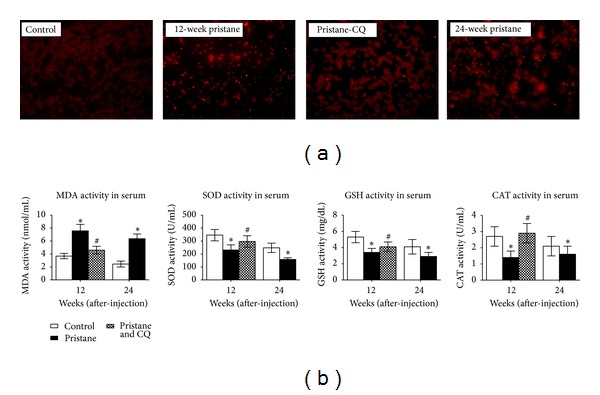
Effects of chloroquine on oxidative stress induced by pristane. Pristane increased the content of superoxide (red, DHE stain) and MDA and decreased the levels of SOD, GSH, and CAT, which could be ameliorated by coadministration of pristane and CQ. Original magnification ×400. CQ: chloroquine; MDA: malondialdehyde; SOD: superoxide dismutase; GSH: glutathione; CAT: catalase. *P < 0.05 versus the corresponding control mice, ^#^ *P* < 0.05 versus mice exposure to pristane for 12 weeks.

During phagocytosis, macrophages produce reactive oxygen species (ROS) such as superoxide anion, hydrogen peroxide. To investigate the effects of pristane on oxidative stress, the levels of ROS, MDA, and endogenous antioxidant enzymes (SOD, GSH, and CAT) in serum were analyzed (Figure 4). Mice exposure to pristane for 12 or 24 weeks illustrated dramatically increased superoxide anion as manifested by dihydroethidium fluorescence staining. Meanwhile, the levels of MDA were significantly increased by 1.11-fold and 0.60-fold compared with that in the normal mice. CQ treatment for 12 weeks significantly reduced the MDA concentration. Alternatively, the activity of SOD, GSH, and CAT was reduced following pristane injection and this reduction was attenuated by CQ treatment.

### 3.5. Th1/Th2 Imbalance Induced by Pristane Was Attenuated by Chloroquine Treatment

**Figure 5:**
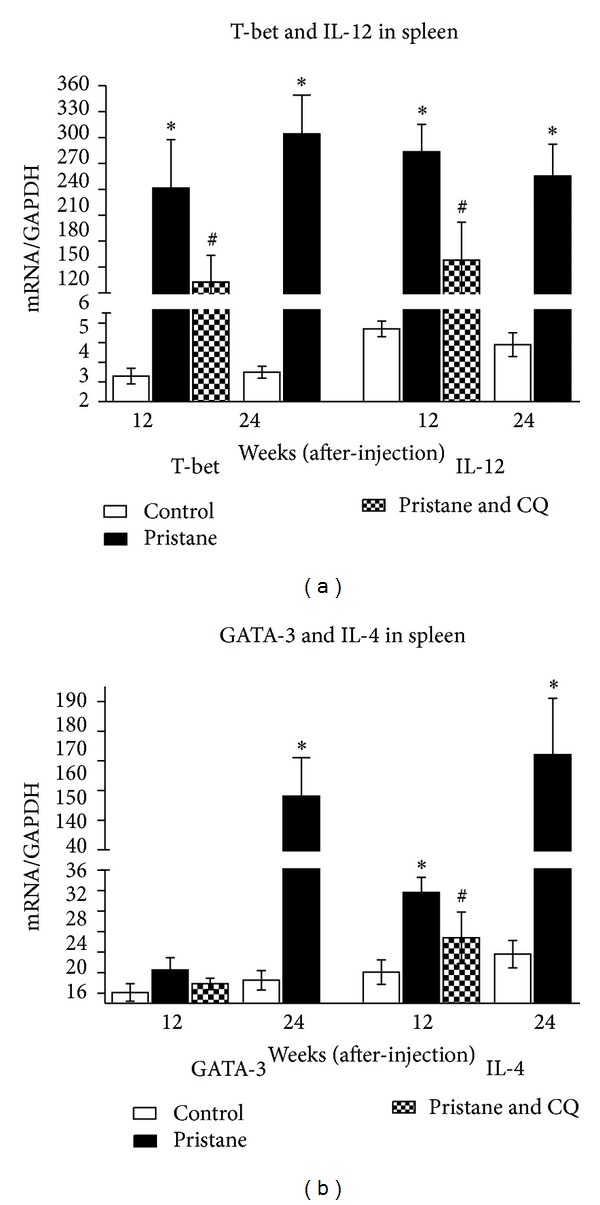
Effects of pristane alone or in combination with chloroquine on Th1/Th2 associated gene expression by RT-PCR analysis. Relative mRNA expression was normalized to GAPDH. CQ: chloroquine. Bars represent mean ± SE of three replicate experiments, *P < 0.05 versus the corresponding control mice, ^#^ *P* < 0.05 versus mice exposure to pristane for 12 weeks.

Considering that Th1 cells have a central role in macrophage activation, we next quantified the expression of Th1- (T-bet, IL-12p35) and Th2-related genes (GATA-3, IL-4) in spleen. At 12 weeks after injection, Th1-associated genes (T-bet, IL-12) were strikingly upregulated by 73.2-fold, 49.7-fold, respectively. While GATA-3 altered insignificantly, IL-4 rose moderately. The ratios of T-bet/GATA-3 and IL-12/IL-10 were 11.91 and 8.11, respectively. At 24 weeks after pristane injection, Th1-dependent gene further increased and paralleled the elevated Th2-related genes. Conversely, the levels of T-bet, IL-12p35, and IL-4 mRNA were decreased following coadministration of CQ and pristane for 12 weeks. Collectively, these data suggested that pristane induced a Th1-biased response at 12 weeks, while it induced a mixed Th1/Th2 gene phenotype at 24 weeks after injection. CQ treatment attenuated the pristane-induced Th1-biased immune response (Figure 5).

### 3.6. Cytokine, IgG2a, IgG1, Ferritin, Fibrinogen, LDH, and Triglyceride in Serum

To gain a better insight into the influence of pristane on cytokine production, the cytokine for IFN-*γ*, TNF-*α*, IL-4, and IL-6 in serum were detected (Table 3). As compared to their corresponding controls, TNF-*α*, IL-6 rose dramatically 12 or 24 weeks after pristane injection. CQ treatment ameliorated these effects as evidenced by a significant decrease of serum IFN-*γ*, TNF-*α*, IL-4, and IL-6 content.

Additionally, as compared with the mice exposure to pristane for 12 weeks, 24-week pristane treatment manifested a 1.58-fold increase in IgG2a and a 2.31-fold increase in IgG1. The IgG2a/IgG1 ratio was therefore reduced by 32.5%, from 8.35 to 5.64. This implied that pristane-treated mice exhibited Th1 skewing at 12 weeks, while they exhibited a mixed Th1/Th2 polarization at 24 weeks. CQ decreased the levels of IgG2a and IgG1 in serum.

Alternatively, substantially elevated anti-Sm, ferritin were found in both 12-week- and 24-week-treated mice. Meanwhile, the levels of LDH and triglyceride were strikingly increased. Conversely, fibrinogen tended to decline at 12 weeks, while it rose marginally at 24 weeks after injection, which reached no statistical significance. Administration of CQ to the pristane-injured mice partly restored these parameters.

## 4. Discussion

The present results firstly demonstrate that a single intraperitoneal injection of pristane triggered macrophage activation syndrome, characterized by hemophagocytosis in spleen and peripheral blood, enhanced lipid phagocytosis by peritoneal macrophages in vitro, erythropenia and leucopenia, and hypercytokinemia, as well as increased lactic dehydrogenase, triglyceride, and ferritin in serum. Concurrently, those mice exhibited enhanced oxidative stress and predominant Th1 immune response. CQ treatment decreased hemophagocytosis and reversed these parameters associated with MAS. These data suggest that MAS can be induced by pristane injection and CQ may be a promising strategy to treat MAS.

### 4.1. Th1/Th2 Polarization

Emerging evidence suggests that CD4^+^T-helper cells can be divided into Th1 and Th2 subsets based upon the cytokines they produce, which are mutually antagonistic. Th1 cells protect against intracellular pathogens, activate phagocytes, induce IgG2a antibodies, and promote delayed-type hypersensitivity responses, whereas Th2 cells protect against extracellular pathogens, activate eosinophils, induce IgE-mediated allergic reactions, and promote other humoral responses in which IgG1 predominates [10]. The Th1/Th2 balance is well known to regulate the immune system under normal condition. Our data indicated that the ratios of T-bet/GATA-3 and IL-12/IL-10 mRNA in spleen, IgG2a/IgG1 in serum at 12 weeks after injection were significantly higher than those at 24 weeks, which suggested that Th1/Th2 shifted from Th1 phenotype at 12 weeks to a mixed Th1/Th2 gene phenotype after treatment for 24 weeks. And our data corroborated the reports that Th1 immune response correlates directly with severity of lupus-like autoimmune disease in MRL mice [11].

Although the molecular mechanism of Th1/Th2 shift in our present study is not investigated, it is reported that the TLR7 downstream signal molecule interferon regulatory factor (IRF) 5 can regulate class switching from IgG1 to IgG2a in B cells [12], while IRF5-deficient mice are protected from pristane-induced lupus via increased Th2 cytokines and altered IgG class switching [13]. Accordingly, it is tempting to speculate that IRF5 is involved in our experimental models to regulate Th1/Th2 differentiation.

### 4.2. Macrophage Activation

The macrophage activation syndrome is a rare but potentially fatal complication of patients with SLE. Naim et al. demonstrated that peritoneal macrophages were activated by pristane injection [14], while our present study further validated these findings. We found exposure to pristane for 12 weeks elicited hemophagocytosis in liver and spleen occurred, renal macrophage infiltration, as well as enhanced lipid phagocytosis in vitro. Additionally, our present study revealed that the levels of ferritin, triglyceride, and LDH in plasma were markedly increased, which further confirmed macrophage activation. Paradoxically, fibrinogen altered insignificantly by pristane treatment. A possible explanation might be a small sample size included in the present work.

### 4.3. Oxidative Stress

Another initial finding in our study was that MDA/SOD ratio was significantly high in pristane-treated mice, indicating enhanced oxidative stress. Macrophages phagocytosis is associated with a burst of respiratory activity which resulted in the production of a variety of molecules and free radicals called ROS, such as superoxide anion, hydrogen peroxide, and hydroxyl radicals. These ROS can damage lipids, proteins, and nucleic acids. MDA levels have been used as a marker for lipid peroxidation. SOD, CAT, and GSH enzymes are important scavengers of superoxide ion and hydrogen peroxide. These enzymes prevent generation of hydroxyl radical and protect the cellular constituents from oxidative damage. Our data demonstrated that pristane evoked an increase in the concentration of blood MDA, indicating oxidative damage to cell lipids. And those results were partly substantiated by Minhas et al. [15] who reported that, in BALB/c mice, levels of ROS and NO in peritoneal fluid were significantly increased 6 months after pristane injection.

### 4.4. Anemia and Extramedullary Hematopoiesis

Hematological abnormalities are common in SLE due to anemia of chronic disease, iron deficiency, autoimmune hemolytic anemia, macrophage activation, and so forth [16]. And the exact cause of anemia in pristane-treated mice cannot be determined at present. In the current study, we demonstrated that erythrocytopenia, anemia, and leucopenia developed 12 weeks after injection, which were partially restored at 24 weeks. Those data were somewhat at odds with the reports that pristane treatment did not change leukocyte in BALB/c mice [17]. This discrepancy may be due to different murine strains used in the studies. Usually, the counts of myeloid, erythroid, and megakaryocytic lineages cells were all decreased during macrophage activation, while, in our observations, suggested platelets were dramatically higher during macrophage activation. This seemingly contradictory result may be due to short half-lives of platelet, predominantly increased megakaryocyte precursors, and a secondary response to accelerated production of red blood cells during extramedullary hematopoiesis [18]. Notably, the reticulocyte counts were increased at 24 weeks after treatment. Since reticulocytes are the products of both medullary and extramedullary erythropoiesis, increased reticulocyte counts may be a compensatory mechanism due to extramedullary hematopoiesis [19].

### 4.5. CQ Inhibited MAS

Chloroquine is a lysosomotropic agent that prevents endosomal acidification. It accumulates inside the acidic parts of the cell, including endosomes and lysosomes. This accumulation leads to inhibition of lysosomal enzymes that require an acidic pH and prevents fusion of endosomes and lysosomes. Chloroquine is commonly used to study the role of endosomal acidification in cellular processes, such as the signaling of intracellular TLRs [20]. Moreover, chloroquine inhibits autophagy as it raises the lysosomal pH, which leads to inhibition of both fusion of autophagosome with lysosome and lysosomal protein degradation [21]. Our present study reveals that CQ treatment decreases the levels of proinflammatory cytokine, which agrees with the reports that CQ is effective in the treatment of diseases associated with increased release of proinflammatory cytokines in SLE [22].

In summary, our study revealed pristane-induced macrophage activation, oxidative stress, and Th1/Th2 skewness in C57BL/6J mice, which was prevented by chloroquine treatment. To our knowledge, this is the first study to report that pristane can induce macrophage activation, oxidative stress and Th1/Th2 skewness. This finding has important implications for the study on macrophage activation syndrome in SLE. However, several limitations to this study need to be acknowledged. Given that the animal model is not fully representative of the human disease, therefore the results observed must be interpreted with caution. Additionally, due to the complexity in the pathogenesis of macrophage activation and Th1/Th2 differentiation, the exact molecular mechanism thus warrants further investigation.

## Figures and Tables

**Table 1:** Primers for real-time quantitative RT-PCR analysis.

Gene	Forward sequence (5′ to 3′)	Reverse sequence (5′ to 3′)
T-bet	AGCAAGGACGGCGAATGTT	GTGGACATATAAGCGGTTCCC
IL-12p35	CACCCTTGCCCTCCTAAACC	CACCTGGCAGGTCCAGAGA
GATA-3	AGAGGTATCCTCCGACCCAC	ATGAAGCCGGAGGGTAAACG
IL-4	ACTTGAGAGAGATCATCGGCA	AGCTCCATGAGAACACTAGAGTT
GAPDH	AGGTCGGTGTGAACGGATTTG	TGTAGACCATGTAGTTGA GGTCA

IL-12, IL-4: interleukin-12, interleukin-4; GAPDH: glyceraldehyde-3-phosphate dehydrogenase.

**Table 2:** Hematological parameters.

	12 wks after treatment			24 wks after treatment	
	Control	Pristane	Chloroquine	Control	Pristane
RBCs (10^12^/L)	10.40 ± 1.51	7.62 ± 0.84*	9.37 ± 1.44^#^	10.15 ± 1.73	8.25 ± 1.21*
HGB (g/L)	151.17 ± 10.61	91.41 ± 14.81*	135.74 ± 17.25^#^	145.3 ± 15.4	113.01 ± 11.34*
Reticulocytes (10^9^)	342 ± 81	326 ± 97	307 ± 75	319 ± 64	378 ± 101*
WBCs (10^9^/L)	10.11 ± 3.15	7.15 ± 2.16*	8.64 ± 1.65*	9.84 ± 2.80	8.63 ± 1.51*
Platelet (10^9^/L)	493 ± 125	745 ± 146*	615 ± 94*	508 ± 98	821 ± 163*

RBCs: red blood cells; HGB: hemoglobin; WBCs: white blood cells. *P < 0.05 versus corresponding control mice, ^#^ *P* < 0.05 versus mice exposed to pristane for 12 weeks. Results are expressed as mean ± SE for seven animals.

**Table 3:** The levels of cytokine, IgG2a, IgG1, anti-ds-DNA, anti-Sm, ferritin, fibrinogen, LDH, and triglyceride in serum.

	12 wks after treatment			24 wks after treatment	
	Control	Pristane	Chloroquine	Control	Pristane
IgG2a (mg/mL)	0.61 ± 0.08	5.12 ± 0.71∗	4.02 ± 0.93^#^	0.55 ± 0.04	8.08 ± 1.05∗
IgG1 (mg/mL)	0.49 ± 0.02	0.62 ± 0.09	0.53 ± 0.12	0.60 ± 0.06	1.43 ± 0.21∗
IgG2a/IgG1	1.21 ± 0.12	8.35 ± 0.07∗	7.36 ± 1.06^#^	0.94 ± 0.05	5.64 ± 0.83∗
Anti-ds-DNA (U/mL)	47273 ± 4035	52641 ± 6514	40671 ± 4035^#^	57003 ± 4583	60460 ± 8353
Anti-Smith (ng/mL)	783 ± 85	4673 ± 647∗	1567 ± 205^#^	865 ± 101	7559 ± 811∗
Ferritin (*μ*g/L)	865 ± 101	1205.22 ± 47.28∗	538.17 ± 50.62^#^	129.50 ± 37.19	373.22 ± 52.67∗
Fibrinogen (mg/mL)	1.52 ± 0.07	1.43 ± 0.21	1.46 ± 0.13	1.57 ± 0.09	1.65 ± 0.27
LDH (IU/L)	279.22 ± 10.9	518.31 ± 34.81∗	301.36 ± 42.53^#^	282.73 ± 32.92	651.22 ± 64.29∗
Triglyceride (mmol/L)	0.80 ± 0.07	1.22 ± 0.31∗	1.09 ± 0.27	0.91 ± 0.14	0.78 ± 0.09∗
TNF-*α* (pg/mL)	104.27 ± 28.83	241.55 ± 31.32∗	158.73 ± 30.69^#^	115.97 ± 21.30	272.00 ± 45.88∗
IL-6 (pg/mL)	76.33 ± 18.15	151.78 ± 42.50∗	101.84 ± 22.36^#^	83.77 ± 17.84	291.15 ± 28.93∗
IFN-*γ* (pg/mL)	59.13 ± 13.92	169.43 ± 32.51∗	94.51 ± 11.35^#^	62.77 ± 11.83	43.89 ± 8.21∗
IL-4 (pg/mL)	46.01 ± 7.00	50.23 ± 8.83	43.57 ± 9.31	43.33 ± 7.82	83.11 ± 11.78∗

IgG: immunoglobulin G; anti-ds-DNA: double-stranded DNA antibody; LDH: lactate dehydrogenase. Results were expressed as mean ± SE for seven mice. *P < 0.05 versus the corresponding control mice, ^#^ *P* < 0.05 versus mice exposure to pristane for 12 weeks.
